# Measuring egocentric distance perception in virtual reality: Influence of methodologies, locomotion and translation gains

**DOI:** 10.1371/journal.pone.0224651

**Published:** 2019-10-31

**Authors:** Philipp Maruhn, Sonja Schneider, Klaus Bengler

**Affiliations:** Chair of Ergonomics, Department of Mechanical Engineering, Technical University of Munich, Munich, Bavaria, Germany; McMaster University, CANADA

## Abstract

Virtual reality has become a popular means to study human behavior in a wide range of settings, including the role of pedestrians in traffic research. To understand distance perception in virtual environments is thereby crucial to the interpretation of results, as reactions to complex and dynamic traffic scenarios depend on perceptual processes allowing for the correct anticipation of future events. A number of approaches have been suggested to quantify perceived distances. While previous studies imply that the selected method influences the estimates’ accuracy, it is unclear how the respective estimates depend on depth information provided by different perceptual modalities. In the present study, six methodological approaches were compared in a virtual city scenery. The respective influence of visual and non-visual cues was investigated by manipulating the ratio between visually perceived and physically walked distances. In a repeated measures design with 30 participants, significant differences between methods were observed, with the smallest error occurring for visually guided walking and verbal estimates. A linear relation emerged between the visual-to-physical ratio and the extent of underestimation, indicating that non-visual cues during walking affected distance estimates. This relationship was mainly evident for methods building on actual or imagined walking movements and verbal estimates.

## Introduction

Continuous technological progress renders virtual reality (VR) applications increasingly popular. Fostered by the games industry, head-mounted displays (HMDs) steadily gain in performance. Unprecedented opportunities to design flexible and highly controllable virtual environments make this technology attractive for a broad range of scientific applications, with the investigation of human behavior being a key research area.

Due to the high interest in traffic safety research, pedestrian simulators, displaying virtual traffic scenarios from a pedestrian’s perspective, constitute a common use case. During the past decade, a broad range of simulator setups has been presented, with many of the more recent ones relying on different types of HMDs. Areas of research include the interaction between different road users [1], street crossing behavior [2], or effects of distraction [3].

Since many studies target collision avoidance and thus require the correct localization of objects, understanding depth perception within this specific context is essential. To decide, for example, whether the time is sufficient to cross a street in front of an approaching vehicle, one has to accurately assess both the current distance to the vehicle and the lane width in relation to walking speed. Similarly, estimates of vehicle speed and acceleration at least partially build on an assessment of what distance was covered within a certain time, equally requiring appropriate distance perception. Previous results indicate that the perception of virtual distances cannot be assumed veridical. Collisions, for example, may result from an underestimation of vehicle speeds and an overestimation of inter-vehicular gaps [2].

Since perceptual processes cannot be observed directly, a methodological challenge consists in quantifying perceived distances. To this aim, various approaches have been suggested, ranging from verbal statements to imagined or actual movements [4, 5]. While existing approaches differ in terms of accuracy and space requirements, little is known as to whether they are equally affected by different types of perceptual cues. Since expanding tracking space renders naturalistic walking increasingly feasible, in particular effects of active locomotion seem relevant. Focusing on the influence of associated visual and non-visual cues, the present study aims to compare common methods used to quantify perceived distances.

## Depth perception in virtual reality

Depth perception can be defined as the ability to perceive the volume of objects as well as their relative position in three-dimensional space [6]. Egocentric depth perception thereby refers to the space between an observer and a reference, whereas exocentric distances concern the space between two external objects. In virtual environments, egocentric distances consistently tend to be underestimated [4, 7, 8], whereas [2] reported an overestimation of exocentric distances. Underestimations in particular affect egocentric distances larger than 1.0 m [8]. A relatively constant degree of underestimation between 2.0 and 7.0 m indicates a categorical rather than a continuous increase in distance compression [7].

Regarding the multisensory integration of depth cues, most literature focuses on visual perception and its interplay with proprioceptive and vestibular feedback resulting from active motion [9, 10]. Auditory [11] and haptic cues [12], in contrast, are likely to influence depth perception to some extent, but not necessarily applicable to all virtual environments. In the following, we thus focus on visual, proprioceptive, and vestibular information.

### Visual depth perception

Visual depth perception is based on structural, pictorial, and motion-induced cues [6] (cf. S1 Fig). Structural depth cues refer to physical adjustments and anatomic relations between the two human eyes, including stereopsis, accomodation, and vergence [6]. Pictorial depth cues arise from features of a two-dimensional scene, such as occlusion, shadows, relative size and height in the visual field, linear and aerial perspective, texture gradient, and the arrangement of edges [4, 6]. Motion-induced visual cues, such as looming, optic flow, and motion parallax [4, 6, 13], further facilitate distance perception if either the spectator or objects in the visual scenery move.

Visual cues in VR may differ from physical environments. For stereoscopic displays, a dissociation of accomodation and vergence arises from presenting different images to both eyes, whereas the curvature of the lenses accommodates to the distance of the display [4, 14]. A lack of details may further limit the availability of pictorial depth cues. However, even in a photorealistic virtual environment displayed by a head-mounted camera, distances were underestimated by 23% (in comparison to only 4% in real world [7]). Similarly, visualizing a reference of known length did not result in more accurate judgments [8]. Hence, distance compression cannot primarily be attributed to a lack of visual details in simplified virtual surroundings or the cognitive misrepresentation of physical units.

### Effects of locomotion

Active locomotion in terms of walking interaction seems to counteract distance compression in virtual environments [15–17]. It thereby appears more effective than other measures, such as presenting participants with a real-world reference [17]. Walking experience in a virtual environment was also shown to affect subsequent distance estimates in the physical world [16]: Prior to the walking interaction, estimates in real world were almost veridical, whereas post-interaction measurements increased by approximately 10%. In [18], however, accuracy only increased for distances that were equal to or smaller than those the participants had previously walked and the calibration of depth perception seemed most effective for larger distances.

In case of locomotion, not only visual, but also proprioceptive and vestibular feedback provides information on the distance covered. Investigating effects of optic flow in the absence of non-visual motion cues, [19] noted a persistent underestimation of the simulated distances, with larger deviations occurring at a shorter duration of the simulated movement. Although humans were thus able to interpret optic flow in terms of distance traveled, estimates were biased. Comparing depth perception in virtual and physical environments, [20] found a less pronounced effect of locomotion in VR. While again, virtual motion was inferred only from optic flow, actual walking provided vestibular and proprioceptive feedback in real world, possibly resulting in a higher gain from locomotion [20]. In the absence of vestibular feedback, [21] reported their subjects to rely primarily on visual information when assessing the distance traveled in comparison to a reference. Interestingly, however, they found proprioceptive feedback from cycling movements to enhance estimates, even if incongruent with the distance indicated by vision.

To distinguish the relative impact of different sensory modalities, the ratio between visually perceived and physically traveled distance may be adjusted. For ratios of 0.5, 1.0, and 2.0, [10] observed physical motion to have a stronger impact on distance estimates than visual perception. The authors thus assumed the sensitivity to visual cues to decrease in the presence of physical motion and interpreted their results as an example of sensory capture, with interoceptive cues overriding visual perception in case of conflicting information. For ratios of 0.7, 1.0, and 1.4, in contrast, [22] found estimates for multisensory conditions to range between unisensory conditions, implying that all available information influenced depth perception. They did, however, note a dominance of cues arising from physical movements for active locomotion, whereas visual cues seemed to prevail in passive locomotion. Elaborating on the differences between active and passive movements, they assumed vestibular cues to be more influential than proprioception, suggesting a linear weighted function to account for the integration of vestibular, proprioceptive, and visual cues.

While the previous results suggest locomotion to influence perceived distances via both visual and non-visual cues, [16] found optic flow to be not only insufficient to counteract distance compression in a blind walking task, but also irrelevant when proprioceptive and vestibular feedback were available. Such discrepancies may be related to the modalities used for the presentation and reproduction of distances. [10], for example, observed that participants strongly underestimated the distance of a visual target when walking towards it blindfolded, whereas estimates were relatively accurate when the distance was not presented visually but by passive motion. If distances were only represented visually, in contrast, they were matched relatively closely when simulating optic flow without actual movements. Visual and non-visual cues thus seem to yield specific and possibly even incongruent information. The performance in estimation tasks thereby depends on the agreement of sensory modalities used for encoding and reproducing distances. Hence, although active locomotion has been demonstrated to counteract the distance compression common to virtual environments, its effectiveness may vary for different types of distance estimates.

## Methodologies for measuring depth perception

Because perceptual processes cannot be observed directly, distance estimates require subjects to express a mental state formed previously. Empirical data suggests that the mode of expression affects experimental results. [23], for example, instructed participants to either indicate when they felt the location of a reference had been reached or to adjust the location of this reference to a distance traveled previously. Distances under consideration ranged from 2 to 64 m. For distances beyond 12 m, the authors reported an underestimation of the traveled distance when placing an external object, whereas distances were overestimated when participants judged the moment they reached a given location. This effect was confirmed by a similar study conducted in a non-virtual environment for distances between 8 and 32 m [20].

Reviewing empirical user studies on egocentric distance perception in VR, [4] stressed the importance to acknowledge differences between measuring methodologies. Summarizing applicable methods, they differentiated between verbal estimates, perceptual matching, and visually directed actions. [24] furthermore distinguished visually guided and visually imagined actions based on differences between blindfolded and imaginary actions.

### Verbal estimates

Verbal estimates require participants to indicate the perceived distance in a familiar or visible reference unit [4]. The target can either be visible during the judgment or participants can be blindfolded [4]. While this method does not require any translational motion and is fast and convenient to use, cognitive processing, a misrepresentation of physical measurement units, and prior knowledge might confound the results [4, 7, 24]. Estimates seem to be relatively precise for short distances [4, 8], whereas underestimation is exacerbated by large distances.

### Perceptual matching

In perceptual matching, the size or distance of objects is compared to a given visual reference. With regard to VR, this reference is either virtual or must be memorized [4]. The corresponding action consists in either adjusting the size or distance of the virtual object or indicating the result of a mental comparison to the reference [24]. In the case of perceptual bisection, the midpoint of a distance is indicated, thereby providing information on relative depth perception [24].

### Visually guided actions

Visually guided movements include throwing, walking and reaching as well as triangulated pointing. Common to all these measures is that the target is not visible during the distance quantification. [4] reported visually directed actions to be the most frequent measure of distance perception, with blind walking being particularly common. Although fairly accurate for a broad range of distances, cognitive processes such as counting steps might bias the results if participants are supposed to indicate a distance they previously walked to. To prevent such effects, triangulation tasks require participants to walk to a designated position and to subsequently indicate the assumed location of the object by pointing or stepping towards the corresponding direction [4].

### Visually imagined actions

Visually imagined actions, with timed imagined walking being the most common variant, no longer require participants to actually perform a movement, but to indicate the expected time needed to do so [4]. Again, estimates can be given while the target is visible, as well as after subjects are blindfolded [24]. Just as verbal estimates, visually imagined actions are independent of spatial restrictions. However, estimates have to be compared to individual walking speed, usually measured prior to the actual experiment. Further variance is introduced by differences in the ability to imagine the walking process [4] and uncertainty as to whether participants mentally include phases of acceleration and deceleration.

### Comparison of methods

A number of studies tried to capture the differences between measuring methods. [7] assumed verbal estimates to require a conscious representation, which is subject to systematic distortion. Comparing them to blind walking, they furthermore pointed out that measuring methods might differ in their susceptibility to manipulations, e.g. because subjects payed attention to the ground texture rather than to a horizon when walking. Despite a trend towards higher and thus more accurate estimates for blind walking, however, effects were statistically insignificant. Furthermore, altering the height of the horizon appeared to equally affect both tasks.

[5] evaluated techniques suitable for experiments in limited space, including verbal estimates, timed imagined walking, blind throwing, and blind triangulated pointing. Comparing two recent consumer HMDs (Oculus Rift and HTC Vive) to real-world behavior, they found distances of 2.0, 3.0, and 4.0 m to be underestimated by an additional 17% on average in comparison to real world. However, distances were also underestimated in real world and distinctive patterns emerged for the four methods. While timed imagined walking, for example, produced severe underestimations of more than 40%, the HMDs matched real-world performance relatively well in this case. Blind throwing in VR, in contrast, showed a generally moderate underestimation, albeit scoring far from real-world performance. Additionally, blind throwing and verbal estimates suggested the most severe underestimations for the farthest distance of 4.0 m, whereas blind pointing was least accurate for the closest distance of 2.0 m.

[24] compared timed imagined walking, verbal estimates and triangulated blind walking in a real-world outdoor environment, a tiled display wall, and a CAVE. While in all environments, timed imagined walking and verbal estimates provided similar results for distances between 2.0 and 12 m, triangulated walking seemed accurate in real-world environments only. In [25], perceptual matching provided different estimates for two virtual environments, whereas results for blind walking and verbal estimates did not reach statistical significance. Comparing verbal estimates and blind walking in real world and in the HTC Vive, only verbal estimates were less accurate in VR. The authors suggested that different perceptual cues influence different types of measures and that participants’ strategies depend on the task to perform. To avoid the latter effect, they recommended to inform the participants about the nature of the respective task after the object was concealed, thus a mental representation had already been formed.

While it is possible that the expectation of a particular task causes subjects to focus on specific cues, it seems just as reasonable that different tasks rely differently on available information. For example, [25] found blind walking and size judgments to be affected by walking interaction, whereas verbal estimates were not. Consequently, when required to express the perceived distance by means of a number, participants did not seem to profit from the additional information provided by locomotion. On the other hand, effects may have been attenuated by cognitive processing: Recognizing that distances before and after the walking interaction were equal, people might have been reluctant to change their initial response.

## Research questions and hypotheses

Previous research demonstrates that, although there is reason to believe that active locomotion counteracts distance compression in VR [15, 17, 22], effects are not equally evident for all measuring approaches [25]. Furthermore, specific methods such as verbal estimates may profit from walking interaction in some cases rather than others [15, 25]. For the technological setup to be used, walking interaction and in particular associated non-visual cues had already been shown to affect verbal estimates [15]. Based on the suggestions by [4], our aim was to investigate whether the observed effects could be generalized to further measures of perceived distance and to clarify the respective role of visual and non-visual cues.

Six methods were compared with regard to the effects of visual and non-visual cues during active locomotion. In addition to verbal estimates, we included visually guided walking, imagined timed walking, blind triangulated pointing, and blind throwing. Visually guided walking differs from blind walking, because, although the target and the surrounding street environment disappeared, reduced visual cues were provided (cf. section Methodology). A sixth method named virtual throwing was based on the concept of blind throwing (for details cf. section Methodology). Estimation accuracy was quantified by means of an error variable based on the ratio of the estimated and the virtually displayed distance (cf. Results). Although relative judgments, referring for example to the equality of distances, may be just as important for traffic safety, our objective was to evaluate the potential of walking to counteract distance compression in virtual environments [7], corresponding to an absolute underestimation.

When comparing methodologies, researchers frequently restrict their selection to methods with limited space requirements [5, 24]. Hence, effects of locomotion are often neglected. Our aim was to evaluate whether previously found differences could be replicated in a virtual city scenario allowing naturalistic walking. A focus was thereby on the comparison of verbal estimates to alternative approaches. Unlike visually directed actions, verbal estimates are often thought to rely on a conscious, typically numerical representation [7] and generally tend to be less accurate at least in comparison to blind walking [7, 25]. If inaccuracy was actually caused by the need for a conscious numerical quantification, one would expect smaller deviations for all other, visually directed actions.

H1 Visually directed actions, which do not require a conscious numerical representation, result in a smaller estimation error than verbal estimates.

Second, we tested for differences between visually directed methods, as have for instance been observed by [5] and [24]. For simplicity and to avoid confusion due to the use of similar but non-identical terminology [4, 24], these methods are also referred to as non-verbal.

H2 The estimation error for different visually directed actions varies.

Third, we expected measuring approaches based on walking movements, such as visually directed and visually imagined walking, to produce particularly low estimation errors if participants had walked to the target previously. This assumption was based on the finding that distance estimates were most accurate if the mode of presentation corresponded to the approach used for distance quantification [10]. For walking interaction, strategies such as counting steps seem for example most helpful if they can directly be linked to the process of distance quantification.

H3 In a scenario in which participants previously walked to the target, measuring methods referring to actual or imagined walking result in a smaller estimation error than other visually directed or imagined methods.

To evaluate specific effects of visual and non-visual cues, we adjusted the ratio between the visually displayed and the physically walked distance. The distance participants walked until they reached the target was thereby scaled for a constant visual distance (cf. section Methodology). The visually displayed distance being equal, lower translation gains corresponded to longer walking, which in turn was expected to result in higher estimates. Based on the known effects of distance compression and previous results [15], we expected lower translation gains to enhance estimation accuracy.

H4 Lower translation gains result in reduced underestimation.

Finally, the effects of translation gains were individually analyzed for the different methodologies. Based on the same assumptions as hypothesis H3, we expected methods referring to actual or imagined walking to be influenced more strongly by non-visual cues.

H5 The estimation error for methods referring to actual or imagined walking is more strongly influenced by the use of translation gains.

## Methodology

### Virtual environment

**Fig 1:**
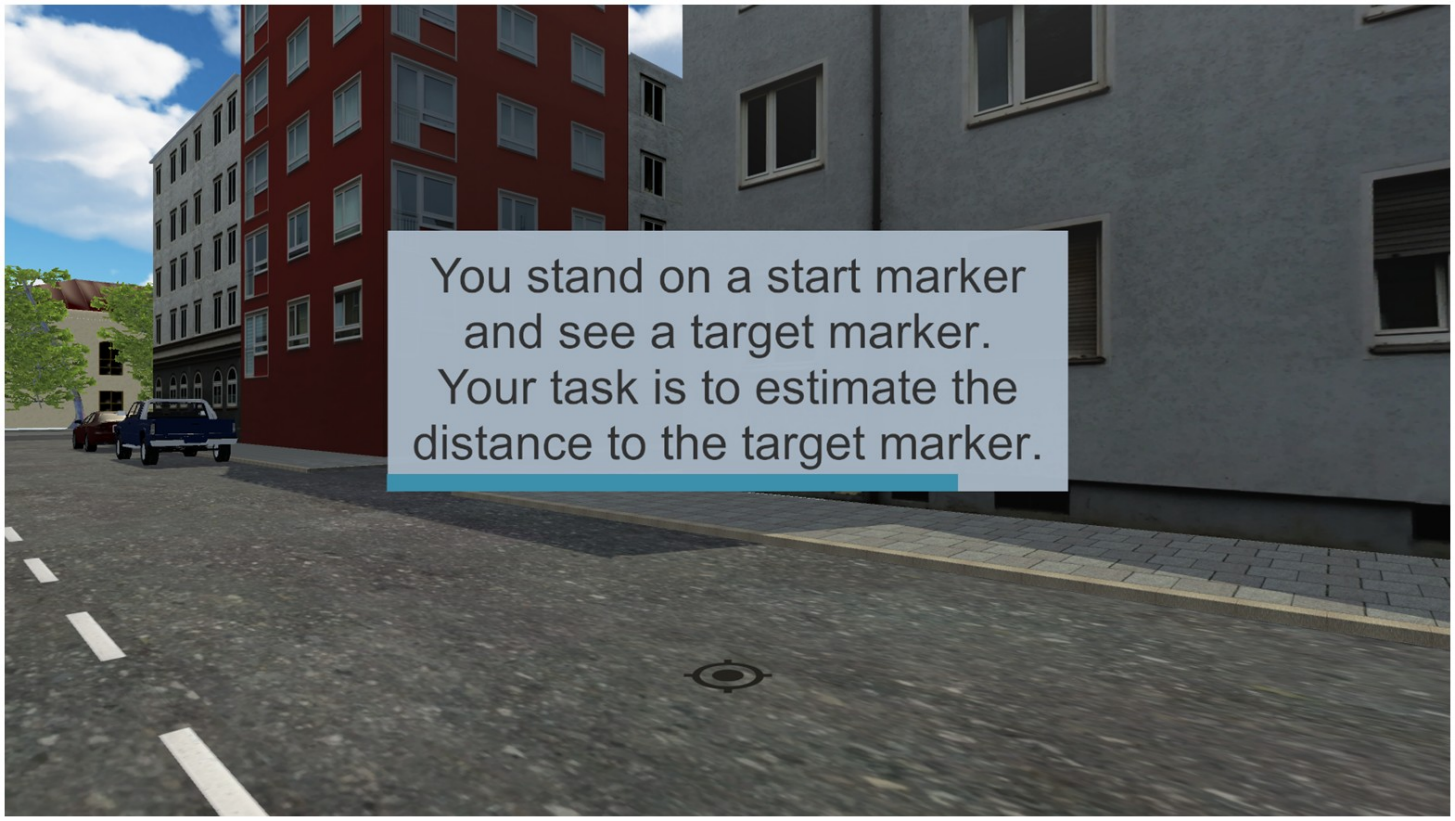
Virtual environment. The virtual environment replicates a typical Munich city street scenario. The environment is rich in pictorial depth cues (e.g. occlusion, shadows, relative size of familiar objects, textures gradients, and linear perspective). The scene shows a target marker to which the participants had to estimate the virtual distance. In the experiment, the written instructions were given in German. The progress bar at the bottom of the text box indicates the remaining time to read the content.

For comparison purposes, the same virtual environment, built in Unity 2017.3, was used as in [15] (cf. Fig 1). The environment was modeled after a typical street in Munich, Germany, and featured a large number of pictorial depth cues (e.g. occlusion, shadows, relative size of familiar objects, textures gradients, and linear perspective represented in VR by houses, parked cars, lane markings, etc.). Seven unique walking tracks were inserted into the virtual environment, and one of them was solely used for practice trials. The six experimental tracks varied in length from 3.0 to 3.5 m (in steps of 0.1 m), and the practice track covered a distance of 4.0 m.

For each trial, the environment was visible for 15 seconds. Afterwards, the street environment disappeared with just a unicolor gray ground layer and the sky remaining. Depending on the measuring approach, additional assisting content was displayed (cf. section Measuring methods).

When a new trial started, subjects were always standing on the positional marker, with the target marker aligned within their sagittal plane. Therefore, no head rotation was needed in order to estimate the distance between the positional and the target marker on the floor. According to the experimental condition, the participants either walked to the target marker and back to the positional marker or remained on the positional marker before estimating the distance. To avoid distraction, no auditory cues were presented. Although the latter can provide distance information [11], this choice seemed justified as the visual scene contained no further traffic participants or other moving objects which would typically emit sounds.

### Equipment

The virtual environment was displayed on an HTC Vive HMD, featuring a dual AMOLED screen with a resolution of 1080 x 1200 pixels per eye and a field of view of 110 degrees. The original Chaperone system (visualization of play area boundaries) was replaced by an individual safety mesh system fitted to the experimental room. Thus, whenever the participant approached the limit of the play area (cf. S2 Fig) by less than 50 centimeters, a blue mesh was faded in to avoid collisions with physical objects in the room. The mesh was faded off as soon as the distance to all boundaries was greater than 50 centimeters again. To improve comfort and fit, the HTC Deluxe Audio Strap was used to attach the HMD to the participant’s head. However, due to the lack of auditory cues, no headphones were used during the experiment. The HMD had a wired connection with a cable length of 5 m (plus 1 m from Link Box to PC). During the trials, participants held one of the HTC Vive controllers to enter and confirm distance estimates depending on the experimental condition.

The virtual environment was hosted on a VR gaming PC running on a Intel(R) Core(TM) i7 8700k CPU with 32 GB Ram and a GeForce GTX 1080 Ti graphics card. Since there were no dynamic virtual objects in the scenario, a stable frame rate with a minimum of 60 frames per second was achieved.

The room (cf. S2 Fig) allowed a maximum walking distance of 6.5 m. The experimenter was positioned in one corner of the room. By positioning the PC closer to the center of the play area, it was possible to make optimal use of the HMD’s limited cable length. Two base stations (Valve lighthouse tracking) were used to track the position and rotation of the headset and controller. Contrary to the manufacturer’s recommendation, the lighthouses (connected via sync cable) were set up with a distance of approx. 6.9 m. However, this did not cause any tracking problems.

### Measuring methods

**Fig 2:**
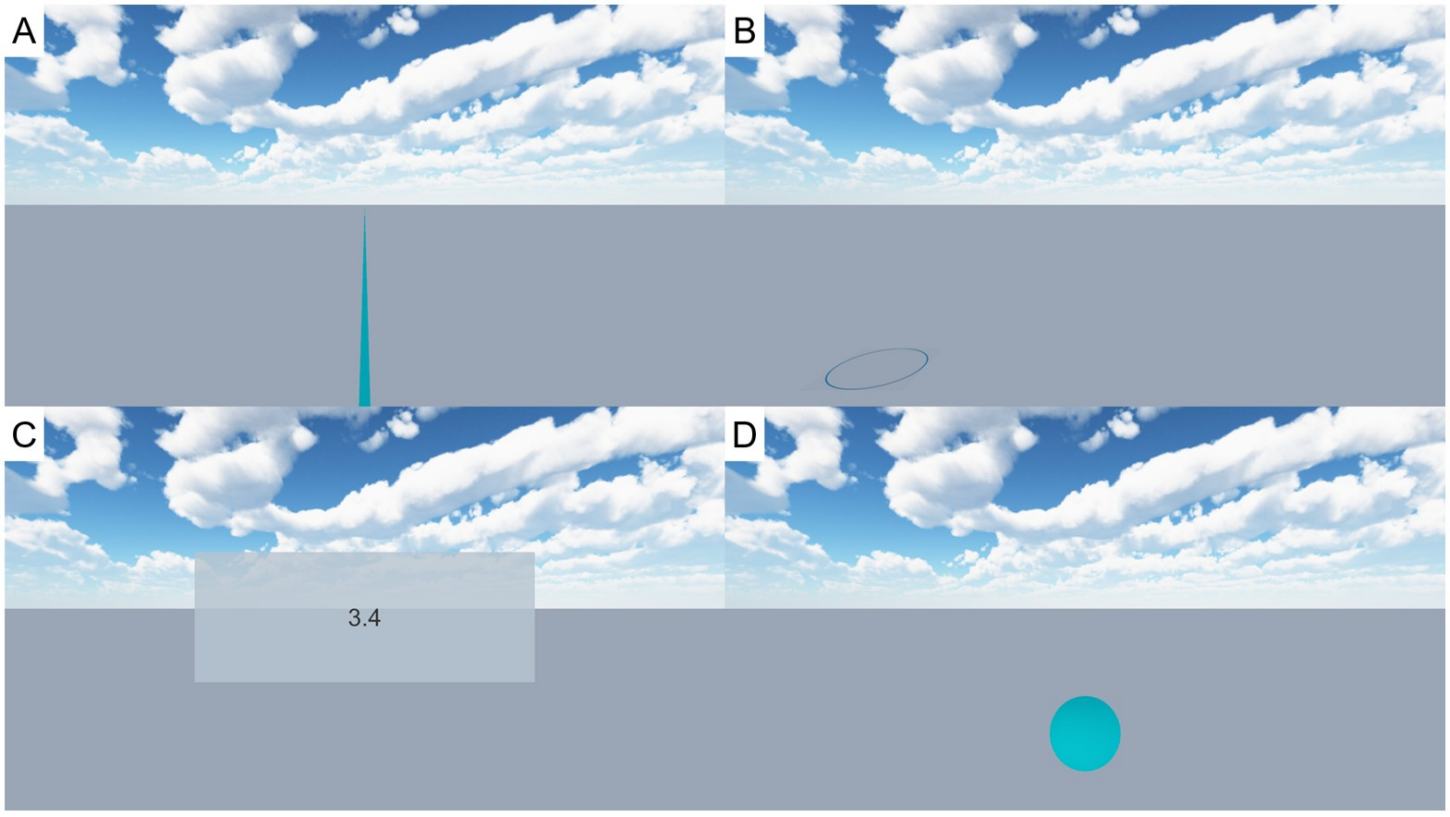
Measuring methods. Depending on the measuring approach, additional content was displayed after the environment was switched off. (A) An orientation line was displayed to guide the direction during visually guided walking. (B) The participant was asked to step into the circle for blind triangulated pointing. (C) A GUI displayed the participant’s verbal estimate after it was entered via a keyboard by the experimenter. (D) The virtual ball the participant had to throw for virtual throwing was also visible during the flight.

After being exposed to the virtual environment, the participant had to express the perceived distance to the target marker. Six different measuring methods were compared. Besides verbal estimates, these included visually directed actions as well as timed imagined walking. The following paragraphs describe the various levels of the factor method. Specific user interfaces are depicted in Fig 2.

#### Verbal estimates

Verbal estimates required the subjects to explicitly verbalize the distance between the positional and the previously viewed target marker in meters with an accuracy of one tenth of a meter (e.g. 3.4 or 5.0 m). Neither the virtual environment nor the target was visible when estimates were given. The experimenter typed in the estimate, which then was displayed in the virtual environment to prevent errors due to miscommunication. If participants acknowledged the input, the experimenter confirmed it.

#### Visually guided walking

The visual reference was turned off and the participant was asked to walk the estimated distance [4]. Instead of blindfolding the participants, in this study, all virtual objects disappeared with only the horizon remaining, separating a unicolor gray floor from a generic sky box. In addition, an orientation line of infinite length was displayed in contrast to studies with auditory cues to ensure straight walking [26] and the participants still experienced optical flow during walking. After walking the estimated distance, subjects confirmed by pulling the controller trigger. The safety mesh was active during the whole experiment. Thus, strong overestimations triggered the safety mesh, potentially serving as a visual reference in those cases. Even for the greatest distance (3.5 m) and the smallest translation gain (0.8, cf. section Locomotion and translation gains), however, the safety mesh would only be visible at an overestimation of 40% onward.

#### Imagined timed walking

Participants had to imagine walking to the target (one direction). They were instructed to hold the trigger button for the duration of the imagined walk. This time measurement was then multiplied by the individual walking speed measured prior to the experiment to calculate the estimated distance.

Typically, the individual walking speed is determined by asking the participants to walk a certain distance, often several times and possibly instructing subjects to walk at a comfortable pace [5, 26–28]. Since in this study, distance perception was investigated solely in VR, the same was true for the measurement of individual walking speed. Therefore, participants were instructed to cross a visible target line at a comfortable walking speed. Walking speed was calculated as the average of four trials. The four measurements comprised two different distances, 3.0 and 3.5 m, representing the minimum and maximum distances employed in the present experiment when neglecting the translation gain. Each distance was walked once in both directions while the virtual environment was visible. The time was automatically started and stopped as soon as the participant crossed the invisible trigger boxes. The visible cyan-colored lines were 0.2 m from the trigger boxes to ensure that participants crossed both measurement points.

#### Blind triangulated pointing

Participants were instructed to look at the target, step to the side into a circle depicted on the floor, and point the controller towards the target. Out of the controller’s lateral translation (Δ*x*) and yaw (*γ*), the intercept (*i*) between the pointed line and the original line between participant and target can be calculated by Eq 1.

$$\begin{array}{c} i=\text{tan}(\frac{\gamma *\pi }{180^{{}^{\circ}}})*\Delta x \end{array}$$ (1)

Various adaptations have been reported for blind triangulated pointing. [24] instructed participants to look at the target, turn by 90°, look again at the target, close their eyes, walk 2.5 m and then point to the assumed position of the target. [5] and [29] exposed participants to the target, then asked them to close their eyes, take two steps to the left and point at the target without visual feedback about the pointing.

In order to control the lateral translation in the present experiment, participants were asked to step into a circle displayed on the floor 1.5 m to the left of the original position. They then pointed at the imagined position of the target marker, confirmed by pulling the controller’s trigger and returned to the original position again marked by a blue circle. There was no visual feedback indicating the pointing direction.

#### Blind throwing

Blind throwing was implemented according to [5]. Participants threw a bean bag (120 g) to the assumed position of the target marker. Velcro attached to the bean bag prevented it from rolling after the first contact with the carpeted floor. The experimenter measured the center position of the bean bag with the help of a second HTC Vive controller. Participants were instructed to throw from below with their strong hand. The estimation was then calculated as the longitudinal and lateral distances between the subject’s position and the measured point.

#### Virtual throwing

Virtual throwing was developed based on the idea of blind throwing, but is independent of an experimenter returning the thrown object and allows for visual feedback of the throwing process in VR. Participants had to pick up a virtual ball with a diameter of 20 centimeters by bringing the controller to the position of the virtual object (floating in front of the participant) and pull the trigger. The ball was attached to the controller as long as the trigger was continuously pulled. With the ball still attached, the participant had to mimic a throwing movement and release the trigger button at the end of the movement to release the ball from the controller. As for blind throwing, participants were instructed to throw from below with their strong hand. The average velocity of the last five frames was passed to the ball at the point of release. SteamVR plugin for Unity—v1.2.3 (Velocity Estimator, Interactable and Throwable) was used for this purpose. On release, the ball was affected by Unity’s standard gravity -9.8 world units (meter) per second squared, aerodynamic drag was ignored. The participants were instructed that the ball would not roll on the floor, thus the first point of contact between ball and virtual ground would be used as measurement. The ball was visible in mid air. In contrast to the other non-verbal methods, participants therefore received feedback regarding their performance, i.e., how far they had thrown the ball. Still, there was no feedback concerning the difference to the actual distance.

### Locomotion and translation gains

In most trials, participants were instructed to once walk to the target marker and return while the environment was visible. The additional visual and non-visual depth cues were thereby subject to the systematic employment of translation gains, altering the ratio between the physical and the virtual distance. Translation gains ranged from 0.8 to 1.2 in intervals of 0.1. A translation gain of 1.0 represents an isometric mapping, where 1.0 m of physical traveled distance is experienced as 1.0 m virtual traveled. For a translation gain of 1.2, in contrast, 1.2 m were virtually passed when physically covering 1.0 m, and for translation gains smaller 1.0, participants had to walk more than 1.0 m to cover that distance in VR. Notably, the length of the experimental tracks ranging from 3.0 to 3.5 m refers to the visually displayed distance, whereas the distance to be walked was adjusted.

A translation gain greater than 1.0 can allow participants to cover longer distances in the virtual environment than possible within the restricted physical space of the experimental room and thus to explore large immersive virtual worlds. While subjective feedback in [30] indicated approval of this method, the gain was of a magnitude that rendered the difference to natural walking obvious. In the current study, in contrast, translation gains were selected to be more subtle in order to influence participants’ depth perception beyond their conscious awareness.

In one trial per method, participants were instructed to experience the virtual environment from a static viewpoint instead of walking to the target marker. They thus had to rely on static visual feedback to estimate the distance, because no information from optic flow, vestibular, or proprioceptive feedback was available specifically with regard to the distance at hand. In contrast to previous studies [15, 25], however, all trials were preceded by walking movements in VR, so general scaling effects are expected to apply to all of them.

### Participants

Thirty university students (age mean 26.3, SD 3.6 years) with an equal distribution of males and females were recruited. Participation required normal or corrected-to-normal vision and no prior experience in a study investigating depth perception in VR. Participants were not reimbursed in any form. They were asked about possible visual impairments and instructed to wear any visual aids under the VR glasses.

### Experimental procedure

S3 Fig summarizes the study protocol as a flow chart. After providing informed consent, the subjects’ interpupillary distance (IPD) was measured. For this purpose, subjects centered a measuring template on their nose from which the experimenter read the IPD. After answering demographic questions referring to gender, age, height, and visual impairments, the subject put on the HMD and adjusted the straps for a firm yet comfortable fit. The IPD of the glasses was adjusted according to the previously measured distance. All further instructions were given via text content windows, superimposed on the virtual environment.

At the beginning, subjects were instructed to walk around for two minutes and get familiar with the virtual environment. To avoid collisions with physical obstacles or walls, the area available for walking was surrounded by the virtual mesh described in the section Equipment, fading in whenever the subject approached the boundaries of the walkable area. To measure the individual walking speed, subjects were asked to cross two virtual lines, displayed on the virtual ground, at a comfortable pace. This procedure was repeated twice for two distances (3.0 and 3.5 m), resulting in a total of four measurements.

During the practice phase, subjects were exposed to the practice track six times. They were virtually translated to a positional marker and rotated towards the target marker. For half of the practice trials, subjects were instructed to walk to the target marker and back, whereas in the remaining trials, they were instructed to solely rely on static visual information in order to estimate the distance to the target marker. In all cases, the environment was visible for 15 seconds. Afterwards, one of the six measuring methods was repeated three times without displaying new virtual content in between. The repetition was chosen after participants in a pre-test had expressed the wish to practice both virtual throwing and blind throwing and it was extended to all methods for comparability and to ensure that instructions were understood. No target markers were displayed during this process in the practice phase or in the trials. The virtual scene was faded black every time the environment was toggled or the subject was re-positioned and then faded in to avoid simulator sickness. During practice, the order of measuring methods and walking interaction was constant for all 30 participants.

Subsequent to the practice phase, subjects had the opportunity to ask questions. They proceeded to the experimental phase by pushing the controller’s trigger button. In contrast to the practice phase, only one distance estimate was given per trial. For each method, six trials were presented, including the five different translation gains and one trial without locomotion. Measuring methods were presented block-wise, with each of the six methods featuring six trials, resulting in 36 trials per participant. Each of the trials within a method corresponded to a different walking track, whose order was randomized. The order of methods was pseudo-randomized across participants to ensure that all possible combinations of methods for the first two positions were realized. Furthermore, the six trials within a method were pseudo-randomized in a way ensuring that for one participant, each method started with a different condition (i.e., one of the five translation gains or the non-walking condition). Completion of the experiment took 30 to 40 minutes, with participants spending approximately 25 to 35 minutes in VR.

The study design aimed to minimize the experimenter’s influence, thus maximizing objectivity. All instructions in the virtual environment were given via text interfaces. Still, on some occasions, the experimenter was consulted to further clarify the instructions.

This study design was approved by the ethics committee of the Technical University of Munich (TUM School of Medicine).

## Results

This study was pre-registered with the Open Science Framework (https://osf.io/69skh/). Methods used for data analysis and inferential statistics were thus predefined with minimal adjustments according to the data and review process.

Inferential statistical tests were carried out using SPSS Version 24 [31] and RStudio [32]. Table 1 gives an overview of the results regarding the hypotheses outlined in the section Research questions and hypotheses.

**Table 1:** Results overview.

Hypothesis		Results	Results without Blind Triangulated Pointing^1^
H1	Visually directed actions, which do not require a conscious numerical representation, result in a smaller estimation error than verbal estimates.	No effect	Lower error for verbal estimates
H2	The estimation error for different visually directed actions varies.	Lower error for visually guided walking compared to blind throwing, imagined timed walking, and virtual throwing	
H3	In a scenario in which participants previously walked to the target, measuring methods referring to actual or imagined walking result in a smaller estimation error than other visually directed or imagined methods.	No effect	Lower error for walking related methods
H4	Lower translation gains result in reduced underestimation.	Reduced underestimation for lower translation gains	
H5	The estimation error for methods referring to actual or imagined walking is more strongly influenced by the use of translation gains.	Significant linear trend for all methods apart from blind triangulated pointing and virtual throwing; largest effect size for walking-related methods	

^1^ Due to relatively large variances of blind triangulated pointing, analysis (in addition to pre-registered tests) has been carried out on a subset excluding these trials for all hypotheses except H5, in which methods were analyzed separately.

In all statistical analyses, estimation error (Eq 2) as a measure of accuracy served as a dependent variable. Overestimation is indicated by negative values and underestimation by positive values. As underestimation appears to be the primary problem in VR, this corresponds to expecting a numerical reduction of the estimation error when distances are perceived more accurately.

$$\begin{array}{c} Estimation\;Error=1-\frac{Estimated\;Distance}{Visually\;Displayed\;Distance} \end{array}$$ (2)

### Walking speed

**Fig 3:**
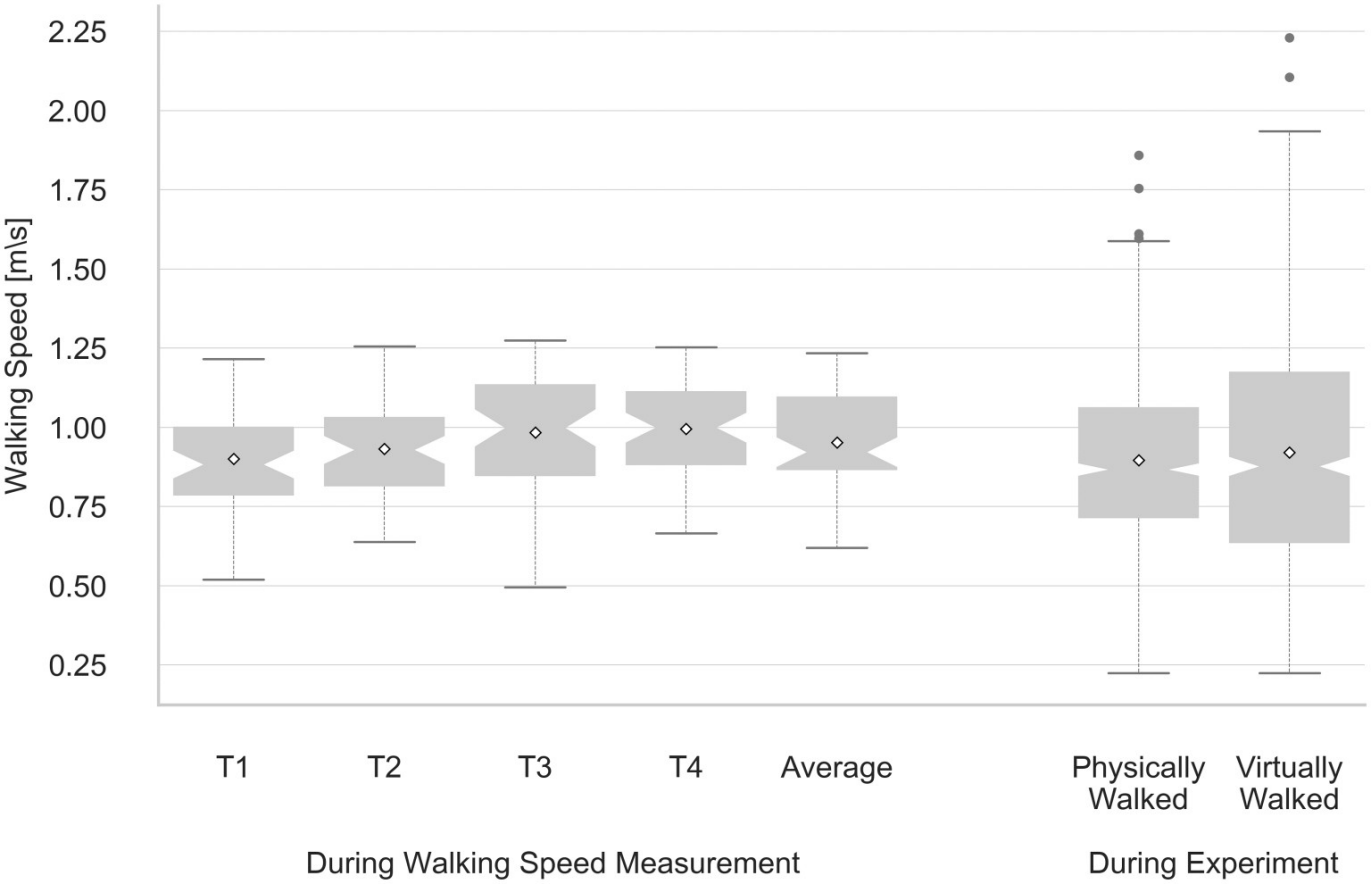
Walking speed results. ’T1’ to ‘T4’ represent the results of the four walking speed measurements, ‘Average’ the arithmetic means of these trials. ‘Physically Walked’ represents the walking speed during the experiment (when participants walked to the target while the environment was visible), and ‘Virtually Walked’ is the virtually traveled distance. Mean values are represented by diamonds, outliers by filled circles. Notches indicate the 95% interval of the median. The whiskers show the range of the data and extend up to 1.5 of the interquartile range.

Fig 3 illustrates the walking speed recorded prior to the experiment for each of the four measurements and on average as well as the walking speed measured during the experiment. The overall mean of the four initial walking speed measurements was 0.95 m/s^2^ with a standard deviation of 0.15 m/s^2^.

For comparison reasons, walking speed was also measured when participants walked to the target marker during the experiment. For a given walking speed in the physical room, the virtual walking speed, i.e. the speed at which movement was displayed in the virtual environment, was thereby affected by the translation gain. Analogously to the a priori speed measurement, the walking speed measurements during the experiment featured a threshold of 0.2 m, i.e., the first and last 20 cm were not taken into account. As in some cases participants did not walk all the way to the target marker but instead stopped before the 0.2 m threshold, only 783 out of 900 walking trials were analyzed. Compared to the initial walking speed measurement, similar values were obtained for the physical (mean 0.90 m/s^2^, SD 0.2 m/s^2^) and the virtual speed (mean 0.92 m/s^2^, SD 0.35 m/s^2^) during the experiment. The larger dispersion in virtual speed indicates that participants maintained their natural walking speed when translation gains were applied, which resulted in a broader variance for virtual speed as it was affected by the translation gain.

### Outliers and data exclusion

Twelve data points concerning nine test subjects were excluded due to technical errors during virtual throwing or the misunderstanding of instructions (participants triggered the controller in blind triangulated pointing before stepping into the circle or did not walk to the target when expected). These participants were excluded from analyses pertaining to the respective data subsets, leaving 21 subjects for the analysis of hypotheses H1 and H2, and 22 subjects in the case of H3, H4 (cf. section Research questions and hypotheses). For H5, in which methods were analyzed separately, the sample size varied between 26 and 30.

Especially for blind triangulated pointing, extreme outliers were observed (cf. S4 Fig), mainly related to one individual. Since a post experiment interview indicated no misunderstanding of instructions, the corresponding data were nonetheless included in the analysis. To simplify graphical interpretation, however, the following figures do not contain outliers. Graphs were created using Seaborn [33] for Python, treating all data points lying more than 1.5 times the interquartile range from the lower and upper quartiles as outliers.

**Fig 4:**
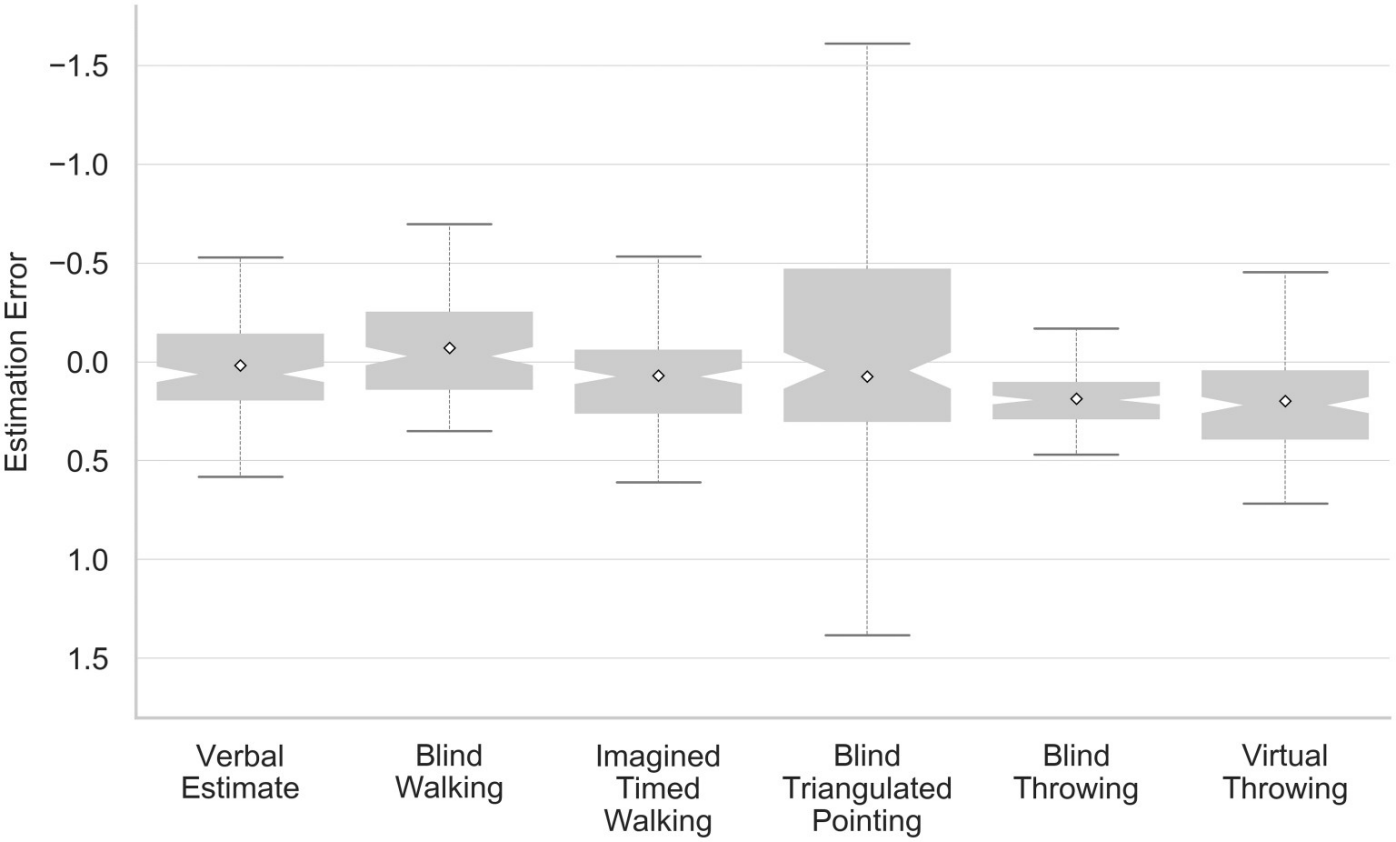
Estimation error for each method over all trials.

The relatively large variance of blind triangulated pointing (cf. Fig 4) may conceal differences between other methods in statistical analyses. Therefore and in addition to pre-registered tests, inferential statistics were also carried out excluding blind triangulated pointing. As certain participants were excluded from the original data set due to missing values for this method, the corresponding subsets featured a sample size of 26 participants for hypotheses H1 to H4.

### Measuring methods

**Fig 5:**
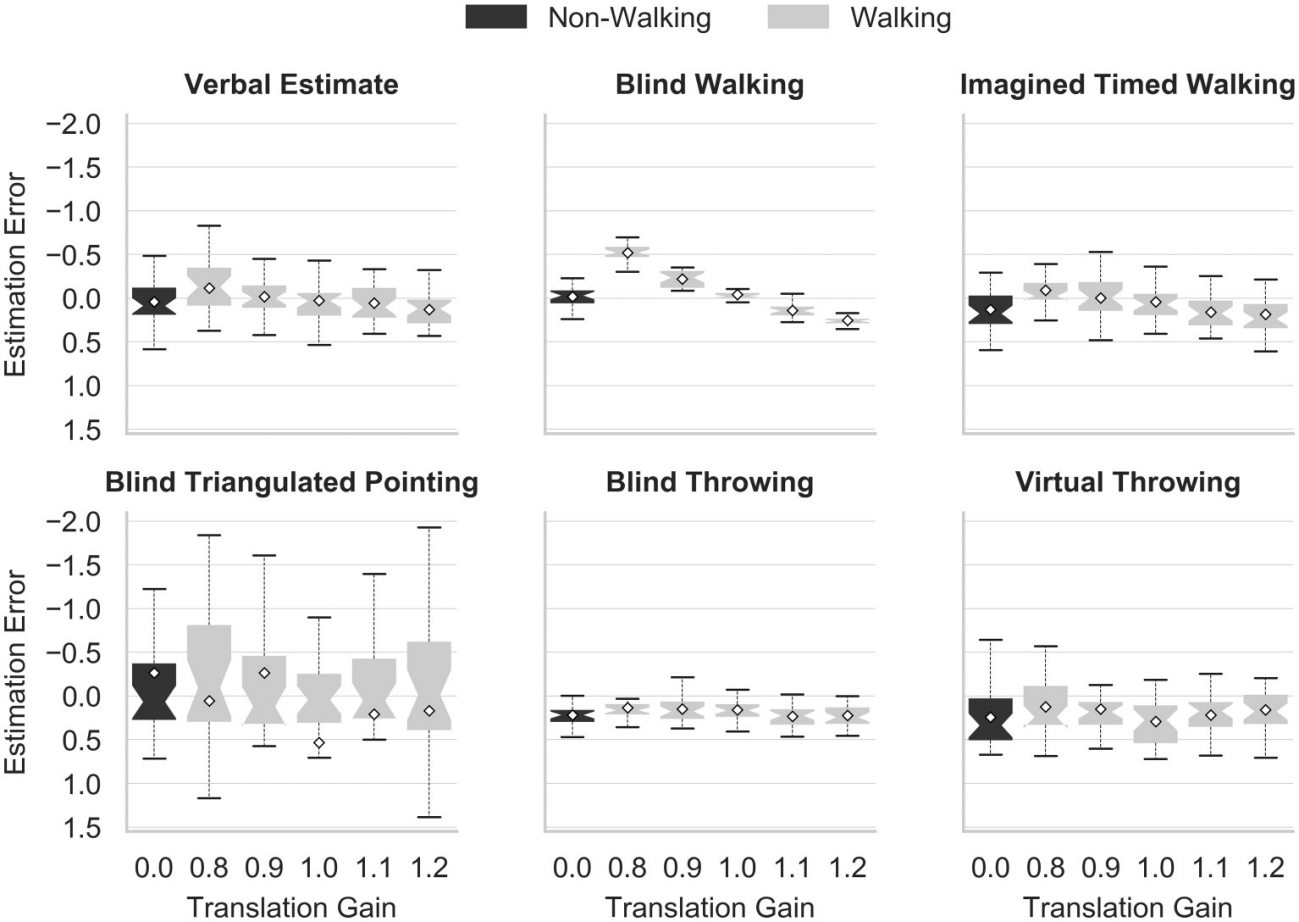
Translation gain factor plot over method. Factor plot displaying each method and the associated influence of translation gain. A translation gain of 0.0 corresponds to non-walking trials.

Hypotheses H1 and H2 concerned differences between the methods, with H1 referring to differences in comparison to verbal estimates and H2 to differences between non-verbal methods. Non-walking trials and the five different translation gains were considered as a combined factor (as each trial either featured a translation gain or corresponded to a non-walking trial). Fig 5 shows values for this combined factor and methods in a factor plot. For the analysis of hypothesis H1, referring to the difference between verbal estimates and non-verbal methods, the data comprised all recorded trials. A Shapiro-Wilk test indicated significant deviations from normal distribution based on a p < 0.05 for all combinations featuring blind triangulated pointing as well as translation gain 0.9—virtual throwing and translation gain 0.9—blind throwing. As those groups were also part of the data set analyzed in H2 and H3, the latter were equally affected. Nonetheless, a parametric contrast analysis was performed, assigning contrast coefficients corresponding to “5” for factor combinations including verbal estimates and “-1” for all other combinations. There was no statistically significant difference in estimation error between the verbal estimates group compared to all other groups (F(1, 20) = 0.257, p = 0.618). The contrast analysis conducted on the data subset without blind triangulated pointing trials featured coefficients of “4” for factor combinations including verbal estimates and “-1” for all other combinations. Results revealed a statistically significant difference in estimation error between the verbal estimates group compared to all other groups (F(1, 25) = 4.735, p = 0.039, *η*^2^ = 0.159). However, in contrast to our hypothesis, verbal estimates on average seemed to result in lower estimation errors than the other methods, thus hypothesis H1 was rejected.

For hypothesis H2, all recorded trials apart from those referring to verbal estimates were included. A 5x6 two-way repeated measures ANOVA was performed including method (5 levels corresponding to five non-verbal methods) and translation gain/non-walking trials (6 levels: 0.8, 0.9, 1.0, 1.1, 1.2, non-walking). A Greenhouse-Geisser correction was applied to translation gain, method, and their interaction based on a violation of the sphericity assumption according to Mauchly’s test (p < 0.001). The mean estimation error showed a statistically significant difference between the non-verbal methods (F(1.137, 22.736) = 5.831, p = 0.021, partial *η*^2^ = 0.226). There was no statistically significant effect for translation gain (F(1.665, 33.3) = 0.89, p = 0.403), nor the interaction term (F(1.689, 33.788) = 0.379, p = 0.653). An ANOVA on the data subset without blind triangulated pointing, in contrast, revealed significant effects for method (F(1.881, 47.017) = 19.934, p < 0.001, partial *η*^2^ = 0.444), translation gain (F(3.696, 92.401) = 35.920, p < 0.001, partial *η*^2^ = 0.59) and the interaction term (F(5.201, 130.015) = 14.095, p < 0.001, partial *η*^2^ = 0.361). Simple effects (calculated in addition to the pre-registration) thereby showed that significant differences between methods only existed for non-walking trials and translation gains smaller than 1.1 (all p < 0.001).

Since sphericity had been violated for method (p < 0.001), Bonferroni-adjusted post-hoc tests were carried out as a robust alternative to Tukey’s HSD post-hoc tests. This adjustment differed from the pre-registered analysis plan. Post-hoc analysis revealed a significantly smaller estimation error (p < 0.01) for visually guided walking when compared to blind throwing (-.264, 95%-CI[-.336, -.192]), imagined timed walking (-.160, 95%-CI[-.283, -.036]) and virtual throwing (-.251, 95%-CI[-.341, -.161]). Post-hoc tests carried out on the data subset without blind triangulated pointing indicated the same results. No pairwise comparisons were carried out regarding translation gains, as the corresponding analysis of a linear trend was examined in the scope of hypothesis H4. Due to significant differences between non-verbal methods, hypothesis H2 was accepted.

Based on hypothesis H3, the estimation error for walking related non-verbal methods (blind and imagined timed walking) was expected to differ from the remaining non-verbal methods. Only trials in which participants walked to the target while the virtual environment was visible were considered. A Shapiro-Wilk test indicated significant deviations based on a p < 0.05 from normal distribution for combinations featuring a translation gain 0.9, 1.0, 1.1, 1.2—blind triangulated pointing, translation gain 0.9, 1.0—visually guided walking, translation gain 0.9—virtual throwing and translation gain 0.9—blind throwing. Still and in accordance with prior testing procedures, parametric statistical tests were chosen. A 5x5 repeated measures ANOVA with the factors translation gain (5 levels: 0.8, 0.9, 1.0, 1.1, 1.2) and method (5 levels corresponding to five non-verbal methods) in combination with a Greenhouse-Geisser correction (based on Mauchly’s test values of p < 0.001) determined no statistically significant effect of translation gain (F(1.202, 25.239) = 1.519, p = 0.234), method (F(1.119, 23.501) = 3.865, p = 0.057), nor their interaction (F(1.188, 24.956) = 0.340, p = 0.603). Greenhouse-Geisser-corrected results based on the data set without blind triangulated pointing revealed a statistically significant effect of translation gain (F(2.942, 73.554) = 46.950, p < 0.001, partial *η*^2^ = 0.653), method (F(1.905, 47.623) = 19.145, p < 0.001, partial *η*^2^ = 0.434) and their interaction (F(4.354, 108.862) = 17.38, p < 0.001, partial *η*^2^ = 0.410). Simple effects indicated that translation gains affected the estimation error for blind throwing, imagined timed walking, and visually guided walking (p < 0.001), but not for virtual throwing. Again, significant differences between methods were only observed for translation gains smaller than 1.1 (p < 0.001).

Differences between non-verbal methods related to walking were analyzed by assigning a contrast coefficient of “3” to factor combinations including visually guided walking or imagined timed walking and a coefficient of “-2” to the remaining three methods. There was no statistically significant difference in estimation error between walking-related and not walking-related non-verbal methods (F(1, 21 = 0.140, p = 0.712). A data subset without blind triangulated pointing was analyzed assigning a contrast coefficient of “1” to factor combinations including visually guided walking or imagined timed walking and a coefficient of “-1” to the remaining methods. Here, a statistically significant difference was found between walking-related and not walking-related methods (F(1, 25) = 49.011, p < 0.001 and *η*^2^ = 0.662). The former generally resulted in a lower estimation error, thus supporting hypothesis H3.

### Translation gains

**Fig 6:**
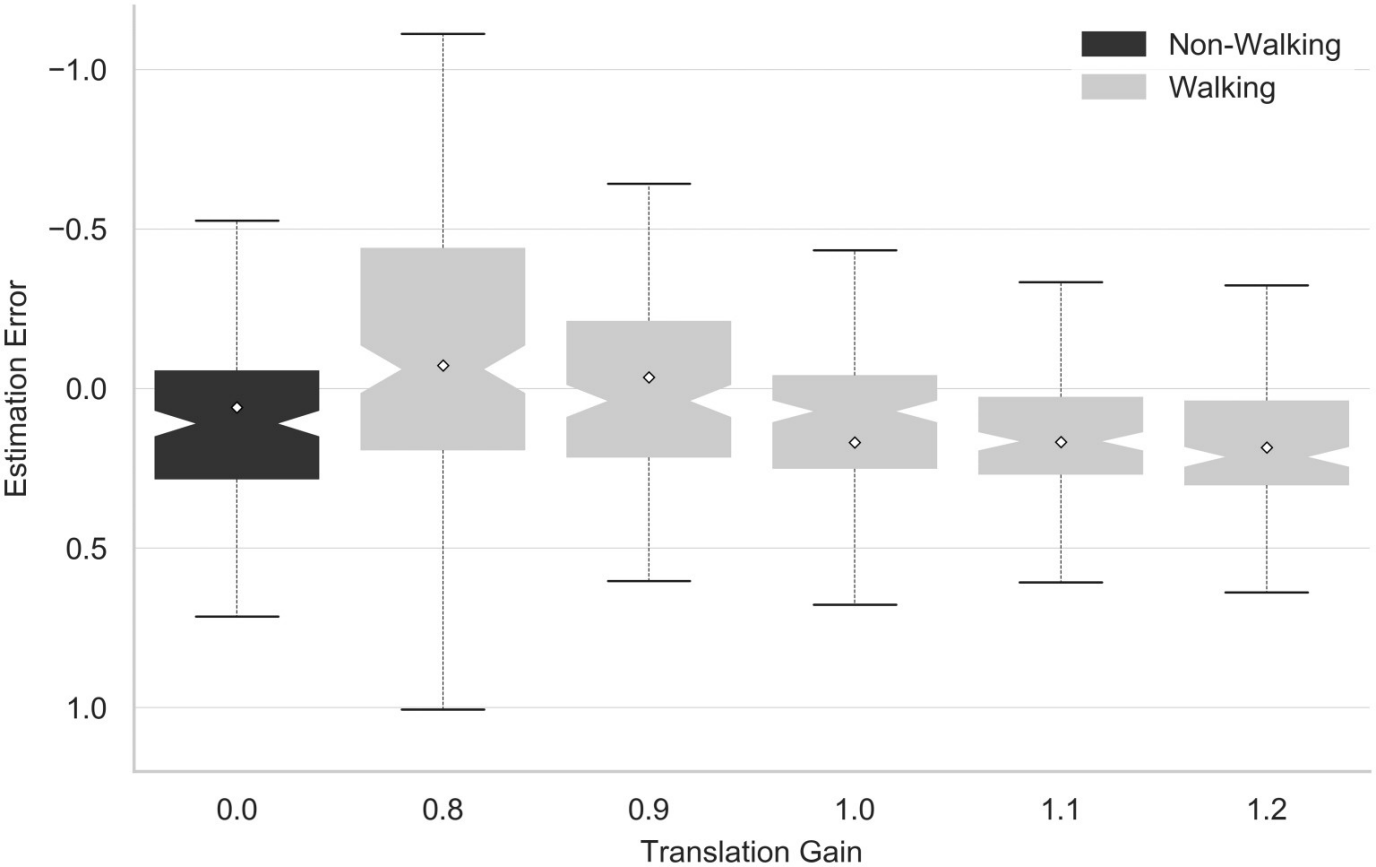
Overall effect of translation gain. Influence of translation gain on estimation error regardless of measuring method. A translation gain of 0.0 corresponds to non-walking trials.

According to hypothesis H4, lower translation gains were expected to reduce the estimation error. Just as in H3, only trials in which participants walked to the target were considered. Fig 6 illustrates the estimation error as a function of translation gain, including the non-walking condition for comparison. A linear contrast showed a statistically significant linear trend (F(1, 21) = 8.032, p = 0.01, partial *η*^2^ = 0.277) with lower translation gains resulting in a lower estimation error, trending towards overestimation for the minimal translation gain of 0.8. The effect size increased for a linear contrast analysis of the data subset without blind triangulated pointing (F(1, 25) = 152.938, p < 0.001, partial *η*^2^ = 0.860). Hence, hypothesis H4 was accepted.

Table 2 outlines the results concerning hypothesis H5, evaluating a possible linear trend of translation gains for each method separately. As stated in hypothesis H5, the estimation error for methods referring to actual or imagined walking was most strongly influenced by the use of translation gains. Hence, hypothesis H5 was accepted. A significant linear trend, however, also produced relatively large effects for verbal estimates and blind throwing.

**Table 2:** Effect of translation gain for each method.

Method	F-value	p-value	partial *η*^2^
Visually guided walking	F(1,28) = 1304.269	< 0.001	0.979
Imagined timed walking	F(1,27) = 141.297	< 0.001	0.840
Verbal estimate	F(1,29) = 80.457	< 0.001	0.735
Blind throwing	F(1,29) = 22.082	< 0.001	0.432
Virtual throwing	F(1,28) = 0.545	0.466	0.019
Blind triangulated pointing	F(1,25) = 0.032	0.859	0.001

### Exploratory analysis—Learning effects

To analyze possible learning effects, a linear mixed model allowing for intercepts to vary between participants was employed using RStudio [32] and the nlme package [34]. Predictors included the trial number within each method, the method itself (dummy coded with verbal estimates serving as a reference) and their interaction term as well as the position of the method in the experimental plan (Eq 3).

$$\begin{array}{c} \begin{array}{cc} EstimationError∼\; & Trial\;Within\;Method*Method\\ & +Method\;Within\;Experiment\\ & +(1|Participant) \end{array} \end{array}$$ (3)

Results (cf. S5 Fig) did not indicate statistical significance (p ≥ .155 for fixed effects) for any of the factors analyzed. Hence, no impact of learning effects across experimental trials was evident.

## Discussion

The present study compared different methods to measure depth perception in VR in the context of active locomotion. The influence of visual and non-visual cues was examined by employing translation gains. Consistent with previous studies [15], an overall underestimation of distances (mean estimation error = 0.079) was observed. However, distance compression seemed less severe than in other cases [4], possibly due to technological enhancements in recent VR goggles [25]. In fact, although earlier studies indicated depth perception in real world to be almost veridical [7, 16], [25] observed an underestimation of a similar magnitude for similar distances in a physical environment.

### Verbal versus non-verbal methods

As verbal estimates may be biased, for example due to an inaccurate mental representation of the reference unit, they were compared to the group of visually directed and imagined actions. In contrast to hypothesis H1, however, verbal estimates did not result in a greater underestimation (i.e., higher estimation error). In fact, the opposite seemed to be true. The mean estimation error for verbal estimates was lower than for all other methods apart from visually guided walking and overall closest to the absolute value of 0 (cf. Fig 5). A mean estimation error of approximately 2% further indicated remarkably lower underestimations in comparison to previous studies [4]. In [15], investigating distances between 3.0 and 4.0 m in the same virtual environment, the estimation error calculated according to Eq 2 averaged between 0.11 and 0.29 depending on the experimental condition. While this could be an effect of the present sample, consisting of individuals with above average capacities, similar demographic characteristics as in [15] render this explanation unlikely. Instead, presenting the distance for a relatively long time might have prevented spontaneous but inaccurate decisions. Alternatively, previous experience with other methods possibly induced carryover effects, which might be examined in future studies.

Importantly, rejecting hypothesis H1 does not contradict findings of higher consistency between real world and VR for blind walking in comparison to verbal estimates [25]. First, the experiment was conducted exclusively in VR, thus no conclusion can be drawn regarding deviations from non-virtual environments. Second, the comparison referred to the group of non-verbal methods and not to any specific approach among them. Nonetheless, the results do not support non-verbal methods in general to be more accurate than verbal estimates.

### Virtual throwing

Virtual throwing was intended as a further development of blind throwing and marks the only method during which visual feedback was provided. During the experiment, numerous participants complained about their performance, mentioning they aimed for a shorter or longer throw. As for all methods, the practice phase comprised only three trials, resulting in a total of nine virtual throws. Although no significant learning effects were confirmed for any of the methods, a weak linear trend towards lower estimation errors can be seen for the virtual throwing averages over the number of trials within the method (S5 Fig). Hence, virtual throwing might benefit from increasing the number of practice trials and improving the behaviors to snap, release, and parse accelerations from the controller to the virtual ball. This might be particularly helpful, since this method has the potential to serve as an alternative for blind throwing which is applicable to experiments with limited physical space and independent of a human experimenter.

### Blind triangulated pointing

In contrast to other studies [5, 29], a relatively large variance was observed for blind triangulated pointing. It is noteworthy that statistical significance concerning the comparison of measurement approaches appeared to be concealed by this noise. Due to the lack of a baseline such as open eyes pointing [29], individual pointing performance could not be compared and the comprehension of instructions could not be validated. However, post-experimental unstructured interviews did not indicate misunderstandings of the test protocol.

In contrast to previous research, the distance that participants had to step to the side was marked by a virtual circle on the ground. While the circle was meant to ensure equal displacement in all trials, it forced participants to look to the floor, thus possibly losing track of the position of the target. In future studies, participants may be guided to a constant position via either auditory signals or visual cues at eye level, enabling them to keep track of the target. Displaying the controller or a virtual light beam might additionally increase accuracy, but also reduce the meaningfulness of the method by providing an additional visual reference which is, for example, absent for blindfolded participants in real-world contexts.

### Walking related methods

Visually guided walking produced significantly lower estimation errors (i.e. closer to 0) compared to imagined timed walking, blind throwing and virtual throwing. Interpreting the non-significant comparison to blind triangulated pointing was complicated by the large variance in this method. Overall, visually guided walking thus seemed to be a particularly precise measure, which was further indicated by the low variance, especially after walking interaction.

Considering the significantly higher estimation error, imagined timed walking does not seem to constitute an adequate replacement for visually guided walking. For imagined timed walking, distance estimates were calculated based on the average walking speed in VR, measured at the beginning of the experiment. While participants were instructed to walk at a comfortable pace, it is unclear if they imagined the same walking speed during the estimation task. The observed physical walking speed during the experiment, however, was even smaller. If participants used this value as a reference, it would thus increase the extent of underestimation. While the cognitive processes involved when imagining ego motion might differ across participants [4], manipulating the translation gain when walking in VR likely affected perceived walking speed. Nonetheless, and perhaps as a result of previous walking, estimates for imagined timed walking were notably more accurate than in [5], who employed distances between 2.0 and 4.0 m.

### Effects of locomotion and translation gains

Neglecting blind triangulated pointing with regard to hypothesis H3, imagined timed walking and visually guided walking resulted in a lower estimation error than the remaining non-verbal methods (i.e., blind throwing and virtual throwing). Graphical inspection and the analysis of hypothesis H5 furthermore suggest both visually guided walking and imagined timed walking to be particularly susceptible to translation gains (cf. Fig 5), pointing to a higher impact of locomotion on walking-related tasks. The adjustment in visually guided walking, however, seems to be considerably more pronounced than for any other method.

The overall linear relationship between translation gain and estimation error was confirmed in line with [15] and according to hypothesis H4, indicating higher estimated distances for lower translation gains. While this finding underlines the effectiveness of non-visual cues arising from ego motion to influence distance estimation, the effect does not seem to affect all methods equally, as indicated by a significant interaction between method and translation gain. While no linear trend was found for blind triangulated pointing and virtual throwing, effect sizes among the remaining methods differed, with effects being particularly pronounced for visually guided walking and large but slightly less obvious for imagined timed walking and verbal estimates. For visually guided walking, the effect of translation gains below 0 even seemed to cause overestimations exceeding the adjustment in walking distance: For a translation gain of 0.8, participants walked 125% of the virtually displayed distance, but estimates referred to approximately 150% of it. Apparently, the mismatch between visual and non-visual depth cues caused participants to overshoot in visually guided walking, not just mimicking the previously experienced locomotion but overcompensating for the translation gain. For the highest translation gains of 1.1 and 1.2, in contrast, differences between methods were insignificant.

## Limitations

The experimental room and dependence on a cable when using an HMD restricted the physical space available and thus the maximal distances to be analyzed. Results cannot be transferred to arbitrary distances, as depth perception has been shown to be influenced by the distance itself [8]. The wired connection to the HMD caused variable tension on the cable depending on the participant’s position in the room, representing a potential additional cue. Similarly, the safety mesh that became visible when approaching the walls might have caused people to stop earlier, in particular for low translation gains and high estimates in visually guided walking. However, visual inspection of the data did not indicate such an effect (cf. S6 Fig).

Participants were not blindfolded before entering the room. Hence, it is possible that a cognitive representation of the available space biased estimates. The latter unfortunately was unavoidable, since, being students of the department, most participants were already acquainted with the room’s dimensions. While a reduction of interpersonal variance might restrict confounding influences such as differences in the comprehension of instructions, the student sample also limits the generalizability to a relatively young age and people with a presumably high technical affinity.

The present study examined depth perception exclusively in VR, lacking a comparison between real and virtual environments. While this seems sufficient to the primary aim of investigating effects of active locomotion on different measuring approaches, it would be desirable to extend the results to real-world contexts.

Finally, the experimental design aimed to minimize participant-experimenter interaction. Despite extensive preliminary tests, occasional questions came up during the experiment. However, it can be seen as one step towards operator-free studies, which besides increasing objectivity might open new ways in participant recruitment for remote and online platforms as commercial VR technology becomes increasingly popular.

## Conclusion

A lot of research has been carried out on depth perception in VR. However, continuous technological advancements, especially in display and tracking technologies, and an apparent dependence on compositional factors [4] require the constant reevaluation of existing knowledge. The present study demonstrated a varying impact of active locomotion on estimates provided by different experimental approaches. In particular, our results confirmed the expected effect of translation gains and indicated varying degrees of susceptibility for different methods. In comparison to previous studies, verbal estimates produced relatively accurate estimates. Hence, investigating the influence of exposure time to the visual target and carryover effects between methods could provide valuable insights regarding the application of this approach. Overall, the results demonstrate considerable differences between a number of non-verbal methods, highlighting the need for more research on differences between measures in general and between visually directed and imagined methods in particular.

## Supporting information

S1 Fig Overview of visual depth cues. (TIF)

S2 Fig Room setup during the experiment. (TIF)

S3 Fig Flow chart describing the experimental procedure. (TIF)

S4 Fig Outliers for blind triangulated pointing. (TIF)

S5 Fig Learning effects. (TIF)

S6 Fig Effect of distance (scaled by translation gain) on visually guided walking. (TIF)
