# Supplementary figures and images for: Measuring egocentric distance perception in virtual reality: Influence of methodologies, locomotion and translation gains
Source: PLoS One. 2019 Oct 31;14(10):e0224651. doi: 10.1371/journal.pone.0224651 (PMC6822760; doi:10.1371/journal.pone.0224651)

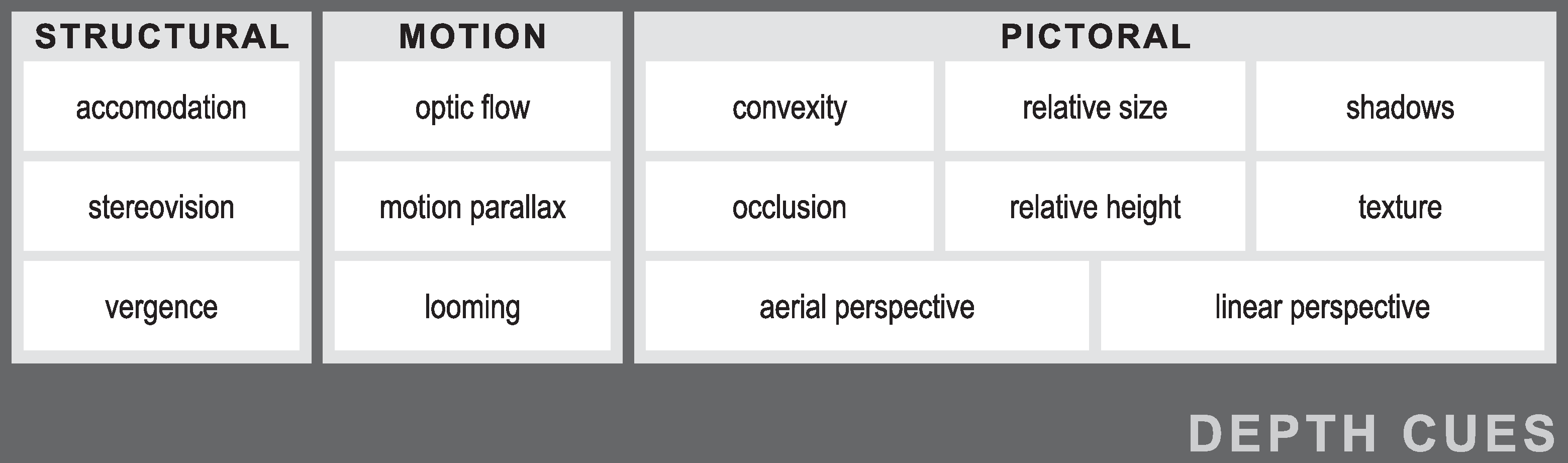

Supplement: S1 Fig — (TIF) [file pone.0224651.s001.tif]

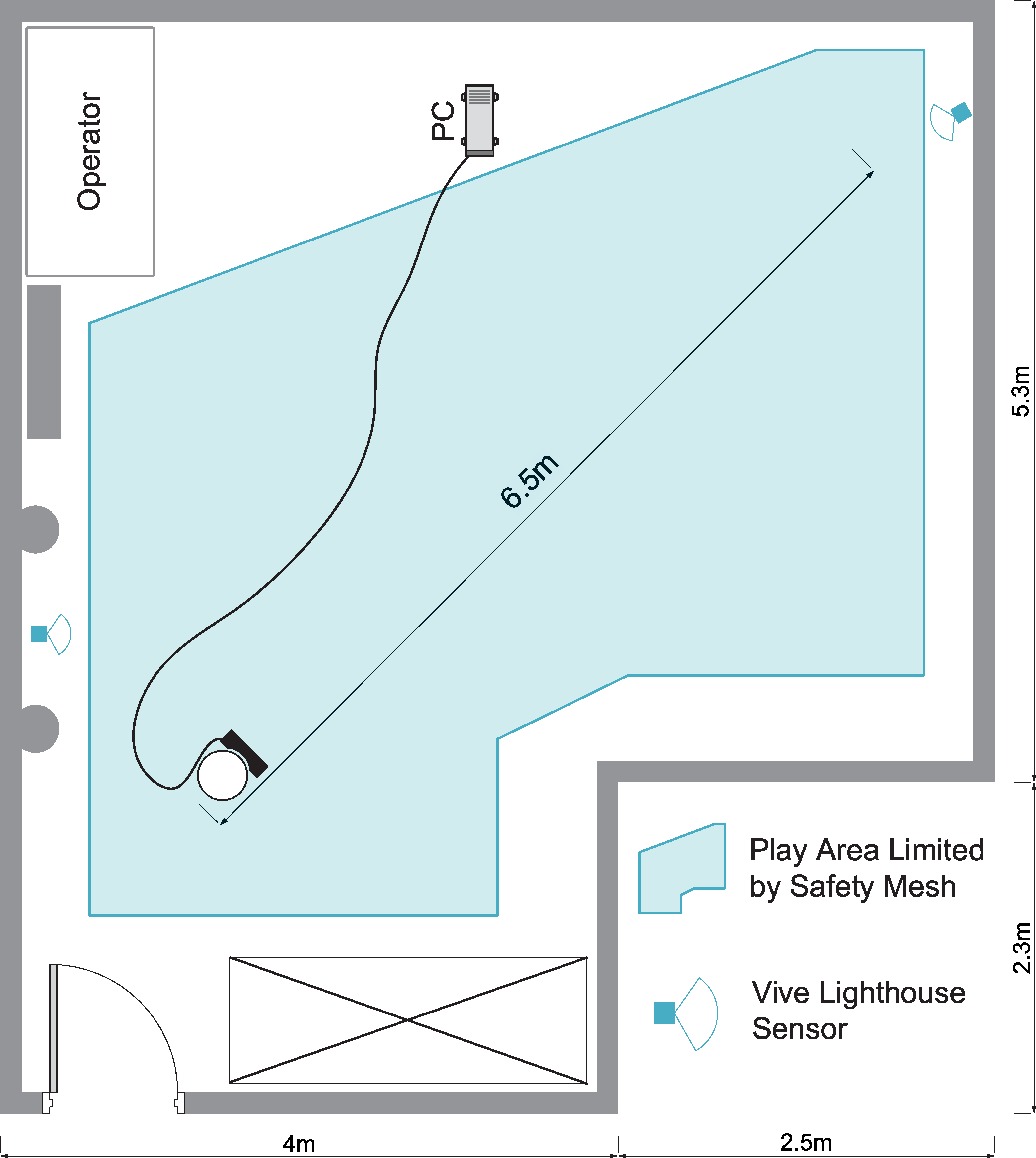

Supplement: S2 Fig — (TIF) [file pone.0224651.s002.tif]

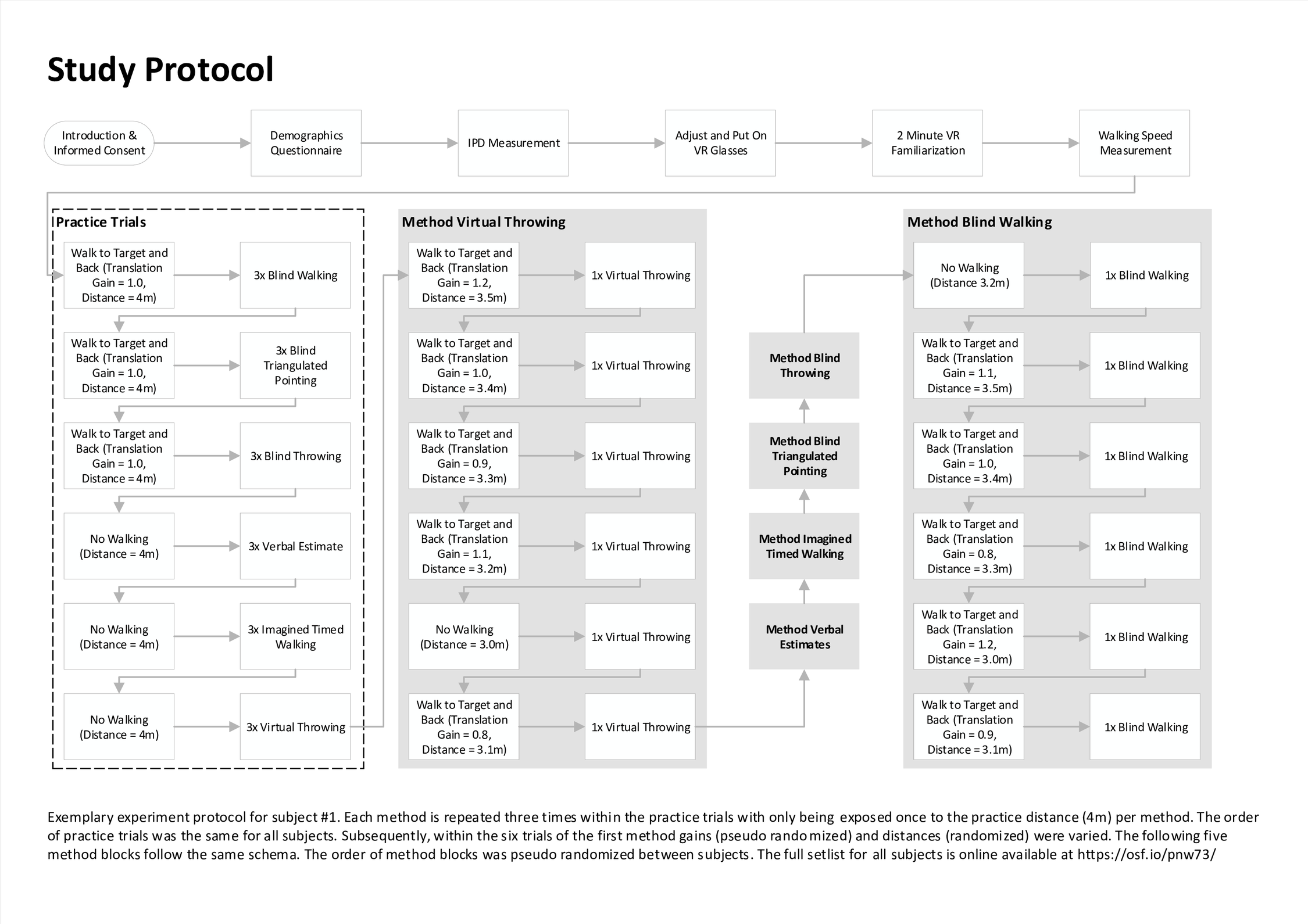

Supplement: S3 Fig — (TIF) [file pone.0224651.s003.tif]

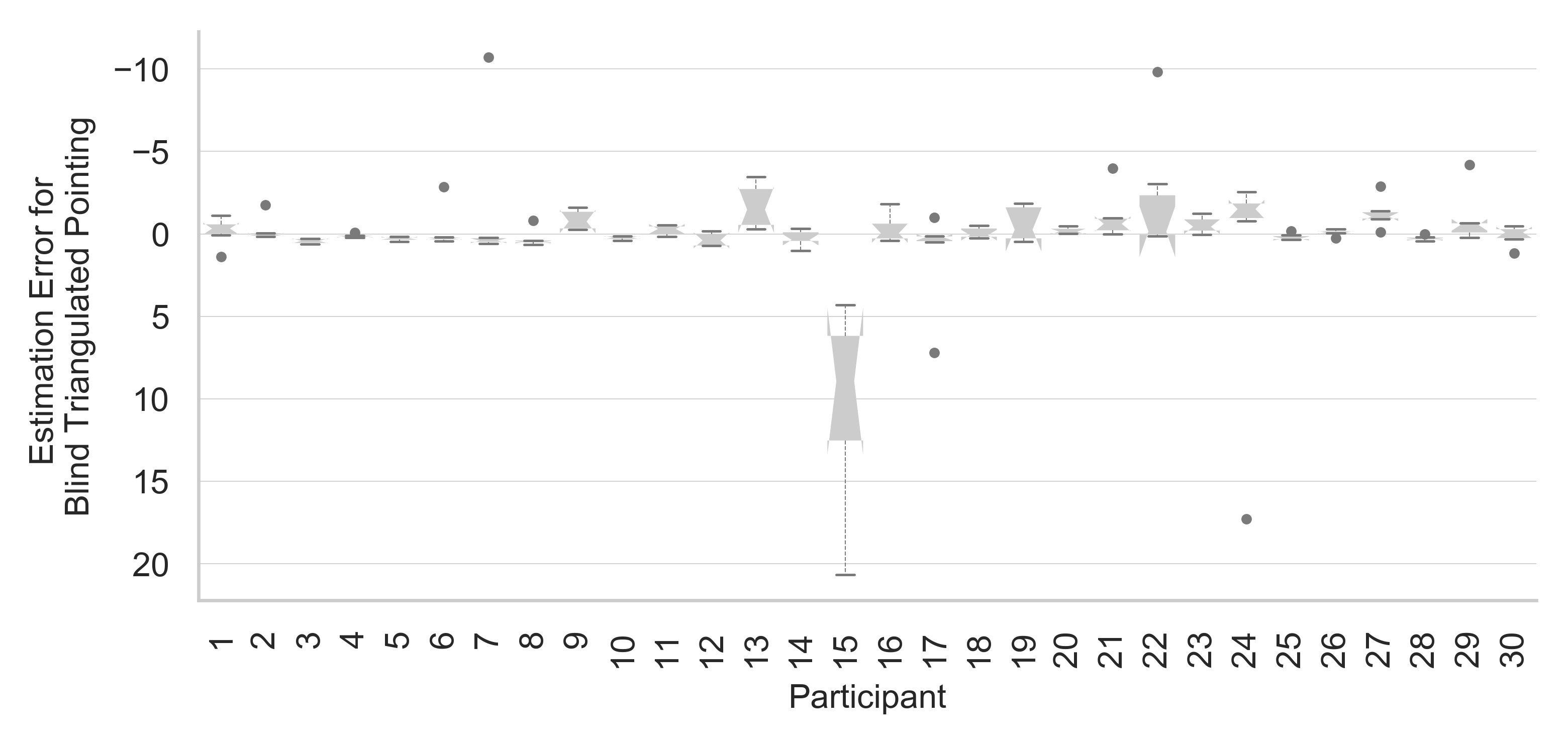

Supplement: S4 Fig — (TIF) [file pone.0224651.s004.tif]

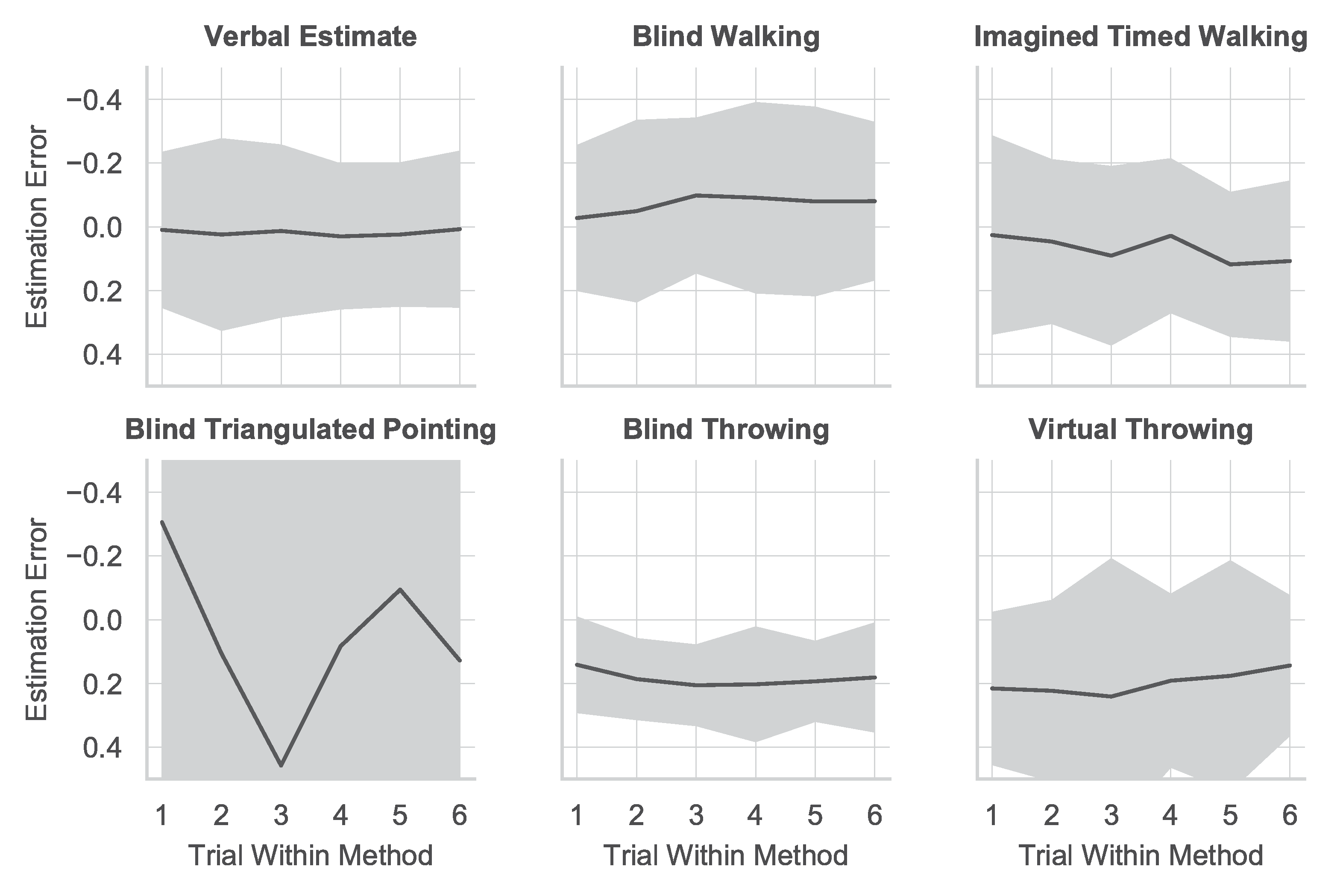

Supplement: S5 Fig — (TIF) [file pone.0224651.s005.tif]

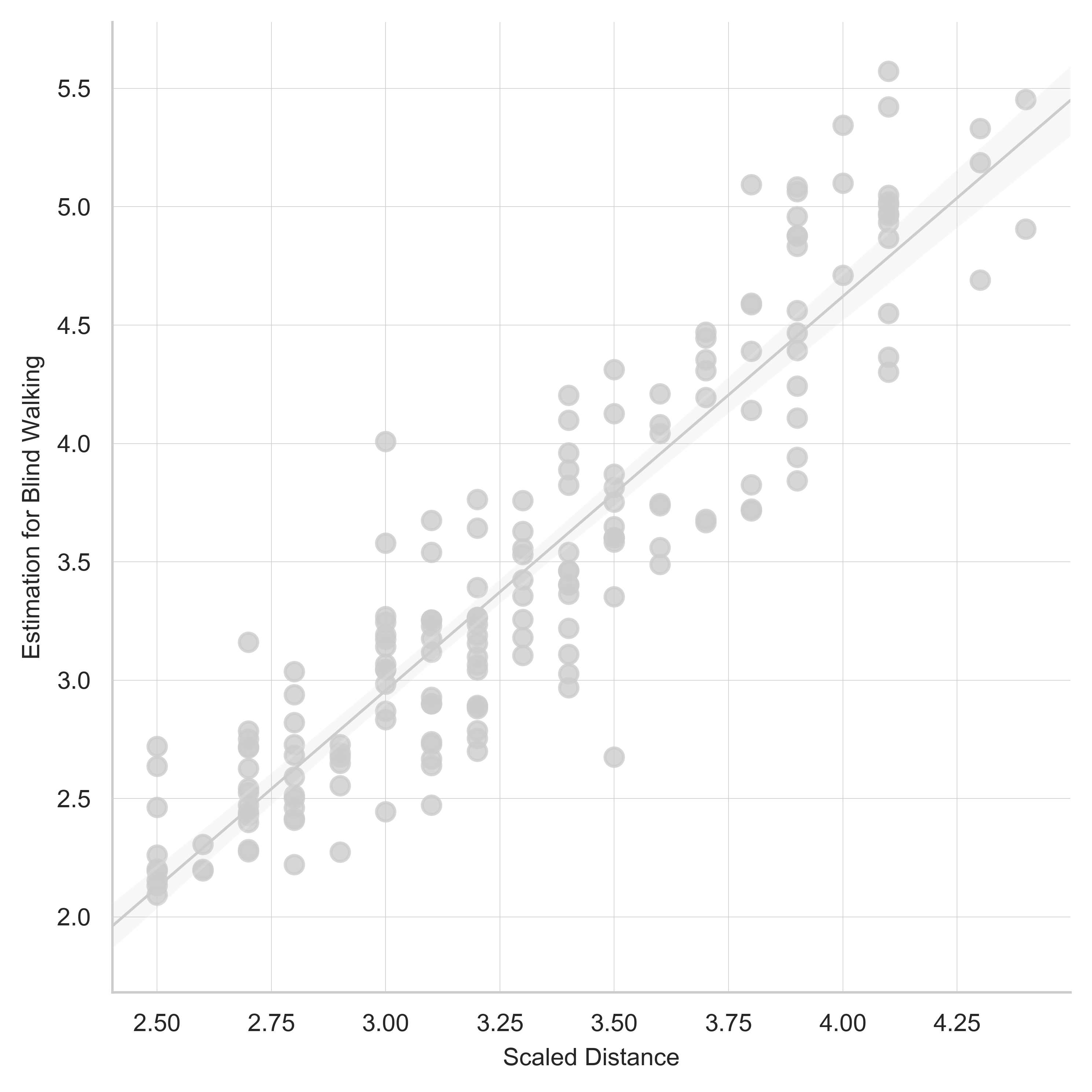

Supplement: S6 Fig — (TIF) [file pone.0224651.s006.tif]
